# Teeth as a Post-Mortem DNA Source for Forensic Parentage Verification in Dogs: A Case Report

**DOI:** 10.3390/vetsci13090948

**Published:** 2026-09-11

**Authors:** Viviana Floridia, Anna Paola Capra, Marco Bitto, Giacomo Oteri, Leonardo Cavallo, Carlo Romano, Adriana Femmino, Gabriele Rea, Vincenzo Cianci, Daniela Sapienza, Luigi Liotta

**Affiliations:** 1 Department of Veterinary Sciences, University of Messina, Viale Giovanni Palatucci, 98168 Messina, Italy; viviana.floridia@unime.it; 2 Department of Chemical, Biological, Pharmaceutical and Environmental Sciences, University of Messina, Viale Ferdinando Stagno d’Alcontres, 31, 98158 Messina, Italy; annapaola.capra@unime.it; 3 Department of Biomedical and Dental Sciences and Morphofunctional Imaging, University of Messina, Via Consolare Valeria, 98125 Messina, Italy; marco.bitto@unime.it (M.B.); giacomo.oteri@unime.it (G.O.); vincenzo.cianci@studenti.unime.it (V.C.); 4 Private Practice, 98100 Messina, Italy; cavaleo72@gmail.com; 5 Sezione Biologia-Reparto Carabinieri Investigazioni Scientifiche, Via Monsignor D’Arrigo, 7, 98122 Messina, Italy; carlo.romano@carabinieri.it (C.R.); adriana.femmino@carabinieri.it (A.F.); gabriele.rea@carabinieri.it (G.R.)

**Keywords:** canine identification, DNA extraction, *post-mortem*, dental DNA, STR genotyping, parentage testing, Staffordshire Bull Terrier, coat color genetics

## Abstract

In *post-mortem* genetic identification, teeth represent one of the most reliable sources of nuclear DNA when other biological samples are unavailable, owing to their ability to protect genetic material from autolysis, microbial degradation, and environmental insults. The present study describes a forensic veterinary case report involving a Staffordshire Bull Terrier litter in which the appearance of blue-coated offspring from a phenotypically black sire and a blue dam prompted parentage verification. The sire died before formal *ante-mortem* sampling could be carried out. In accordance with Regulation (EC) No. 1069/2009, Article 15, the carcass was interred in the owner’s garden. Before burial, two teeth were collected as the sole biological material available for identification by the official veterinarian, ensuring an unambiguous chain of custody from death certification to laboratory analysis. DNA extraction was performed using two independent protocols: Method A, a silica-membrane-based purification (QIAamp DNA Kit, Qiagen), applied to dental pulp, and Method B, a decalcification-based workflow (T-Bone Ex Kit) followed by DNA extraction using EZ2 Connect Instruments (Qiagen). Quantification of the DNA samples obtained were done by fluorometry. Short Tandem Repeat (STR) genotyping was performed using the Canine Genotypes™ Panel 1.1 by Applied Biosystems (Thermo Fisher Scientific, Waltham, MA, USA) targeting the 18 autosomal ISAG-recommended microsatellite loci. Parentage assignments were carried out against reference STR profiles obtained from peripheral blood of the dam and offspring. The two methods, considering a different biological matrix and different operating conditions, allowed us to obtain the STR profile, but Method B showed quality electropherograms, improving the confidence and interpretation of all the markers analyzed. The differences observed between the two approaches may reflect a combination of factors, including the different dental matrices analysed, the quantity and quality of the starting material, tooth identity and tissue preservation, and differences inherent to the extraction procedures themselves; therefore, in this case, the more complete and clearer STR profile obtained with Method B should be considered a case-specific observation rather than evidence of a direct effect of the extraction method. Overall, regardless of the DNA extraction method used, the results did not exclude the biological paternity of the blue-mantled offspring. Furthermore, this case report provides practical methodological guidance for DNA recovery from canine teeth and supports their use as a reliable source of *post-mortem* DNA for STR-based parentage testing.

## Simple Summary

Teeth can preserve DNA after death and may be useful when *ante-mortem* samples are unavailable. This case report describes a Staffordshire Bull Terrier litter in which blue-coated puppies were born from a black sire and a blue dam, requiring parentage verification. The sire died before genetic sampling, and two teeth were collected before burial as the only available biological material. DNA was extracted using two applied methods: silica-membrane purification (Method A) and decalcification followed by automated extraction (Method B). DNA was quantified by fluorometry, and 19 ISAG-recommended canine STR loci were analysed. Method B yielded a higher DNA concentration compared to Method A (0.3 vs. 0.1 ng/μL). The two methods, considering the application and operational differences, allowed us to obtain the STR profile but Method B showed quality electropherograms, improving confidence in and interpretation of the STR profile compared to Method A. Both methods produced profiles that did not exclude the deceased sire as the biological father. These findings support canine teeth as a reliable *post-mortem* DNA source for parentage testing.

## 1. Introduction

Genetic identification and parentage testing in *Canis lupus familiaris* have become routine instruments in modern dog breeding, dispute resolution, and population management. Since the consensus reached by the International Society for Animal Genetics (ISAG) comparison tests, Short Tandem Repeat (STR) genotyping based on the ISAG core marker panel has represented the international reference standard for canine identification and pedigree verification [1,2]. More recently, in 2020, ISAG released the first SNP-based Canine Parentage and Identification Panel, providing a higher-density, automation-friendly complement to STR-based workflows [3]. STR genotyping nonetheless remains the most widely adopted technology in routine veterinary diagnostic laboratories, owing to its discriminating power on small numbers of markers, robustness on degraded samples, and broad availability of validated commercial kits [4,5].

The demand for canine parentage testing has grown steadily over the past two decades, driven by several practical scenarios. Breeders routinely request internal verifications to confirm assigned sires and dams within their selection programmes, especially when mating designs involve multiple males or natural service in group housing [6]. Kennel clubs and breed registries mandate confirmatory parentage analyses as part of random or systematic herd-book audits, and as a corrective tool in cases of suspected pedigree misattribution [7]. Genetic testing is also frequently required when documentation needed for pedigree issuance is submitted late or incomplete, and when biological evidence is needed to confirm relationships retroactively.

The present case report originated from a breeding scenario involving a Staffordshire Bull Terrier litter obtained from a phenotypically black sire and a phenotypically blue dam, both registered with the Italian Kennel Club (ENCI). After natural mating, the birth of three blue-coated puppies raised questions regarding the expected inheritance pattern, given the father’s black phenotype.

In Staffordshire Bull Terriers, as in domestic dogs generally, coat colour is controlled by the interaction of multiple genetic loci. The blue coat phenotype is associated with the homozygous recessive genotype (*dd*) at the *D* (dilute) locus, encoded by the *MLPH* gene. This gene located on canine chromosome 25 (CFA25; NC_051829.1: 48,506,862–48,555,710) encodes melanophilin, a key component of the melanosome transport complex involved in pigment granule distribution within melanocytes [8,9]. The recessive *d* allele affects melanosome transport, resulting in dilution of black eumelanin into the slate-grey colour phenotype defined as “blue” in canine breeding terminology [10,11,12]. Phenotypically black individuals may carry a single copy of the recessive allele (*d*) without showing dilution but retaining the ability to transmit the allele to their offspring. Therefore, the mating between a *Dd* sire and a *dd* dam is expected to produce both black (*Dd*) and blue (*dd*) offspring according to Mendelian segregation. Since the sire’s genotype at the *D* locus had not been previously characterised and the unexpected coat colour distribution could have raised concerns regarding pedigree attribution, STR-based parentage verification was requested.

The death of the sire from non-infectious causes prevented the collection of conventional biological samples and required the identification of alternative DNA sources for parentage verification.

When STR verification is needed *post-mortem* to authenticate offspring already born from frozen semen, to settle inheritance or legal disputes, or to support conservation programmes for local breeds, the recovery of nuclear DNA from skeletal or dental remains becomes the only viable option [13].

Among *post-mortem* sources of DNA, teeth are recognised as particularly resilient [14]. The mineralised structure of enamel, dentine, and cementum protects the pulpal compartment from autolysis, microbial colonisation, and environmental insults, preserving nuclear DNA over extended *post-mortem* intervals [15,16,17]. However, this same mineral matrix imposes several methodological challenges, including efficient disruption of dental tissues, removal of PCR inhibitors, and recovery of sufficient amplifiable nuclear DNA from limited biological starting material [18,19]. Despite the forensic relevance of these scenarios, studies describing DNA extraction protocols specifically applied to canine teeth, with downstream assessment of STR performance for parentage assignment or identification, are still poorly represented in the veterinary and forensic literature.

In this context, the present study aimed to describe two different extraction protocols on two teeth collected from a deceased Staffordshire Bull Terrier dog: a silica-membrane-based method (Method A) and a decalcification-based workflow followed by DNA extraction using EZ2 Connect Instruments by Qiagen (Method B), evaluating DNA yield and STR genotyping completeness, with a final possible parentage attribution comparing the profiles of the dam and offspring. By considering realistic forensic scenarios involving different types and conditions of *post-mortem* dental tissue, this case report provides practical methodological guidance for DNA recovery from canine teeth and supports their use as a reliable source of DNA for STR-based genotyping.

## 2. Materials and Methods

### 2.1. Case Description and Biological Sample Collection

The present case involved a litter of Staffordshire Bull Terriers obtained from a mating between a phenotypically black sire and a phenotypically blue dam. After natural mating, the dam gave birth to three blue-coated puppies and due to the unexpected coat colour distribution within the litter and the absence of previous genetic characterization of the sire, the owner requested a formal STR-based parentage verification.

The investigation was further complicated by the subsequent death of the sire from non-infectious causes. In accordance with Article 15 of Regulation (EC) No. 1069/2009 of the European Parliament and of the Council, which permits the burial of the carcasses of pet animals that have not died from transmissible diseases under specific conditions, the body of the dog was buried without any type of wrapping in the owner’s garden and covered with lime.

Before burial, two teeth (first (M1) and second (M2) molar) were extracted under aseptic conditions, as the sole biological material available for identification, by the official veterinarian, ensuring an unambiguous chain of custody from death certification to laboratory analysis. The two teeth were stored at −20 °C for two months before genetic identification.

Reference biological samples for the dam and all offspring in the litter were collected by peripheral blood into EDTA-coated tubes and stored at −20 °C until processing.

All analyses were performed in an ISAG-accredited laboratory to ensure the accuracy, reliability, and reproducibility of the analytical data obtained. The study was conducted in compliance with applicable national regulations on the use of animal biological material for research and diagnostic purposes.

The genetic objectives of the study were therefore threefold: (i) to extract amplifiable nuclear DNA from *post-mortem* dental tissues using two independent protocols; (ii) to obtain a complete STR profile of the deceased sire according to the ISAG canine marker panel; and (iii) to perform parentage assignment of the offspring, confirming the biological relationships within the litter.

### 2.2. Method A: Silica-Membrane-Based DNA Extraction from Dental Pulp

#### 2.2.1. Tooth Sectioning and Pulp Retrieval

**Figure 1:**
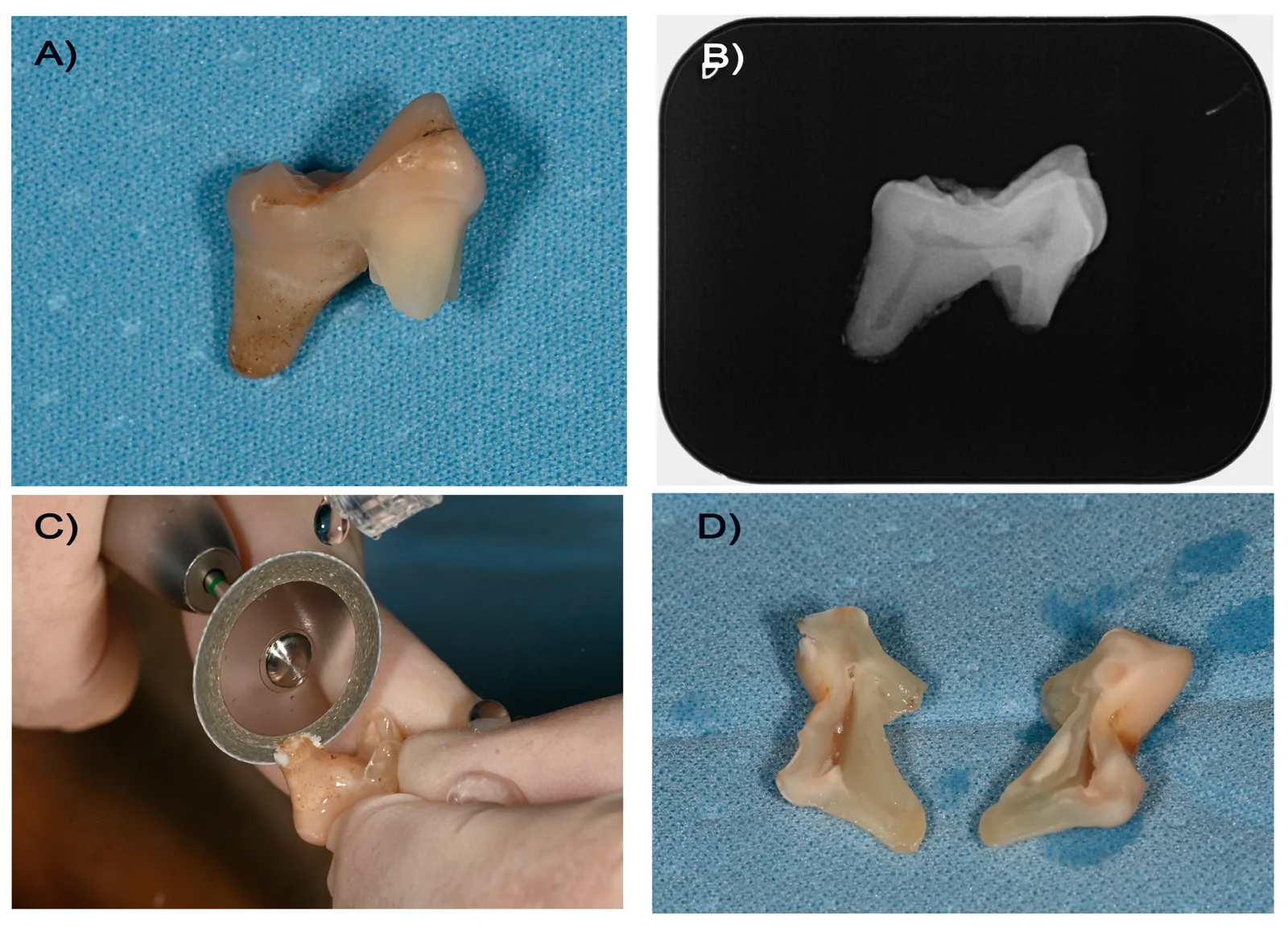
(**A**) Clinical photograph of the intact extracted second molar—M2 tooth before sectioning, shown in the same orientation as the radiograph. (**B**) Periapical radiograph of the extracted tooth acquired to evaluate the root and endodontic morphology (root canal anatomy and pulp chamber) before processing; (**C**) longitudinal sectioning of the tooth along its major axis with a diamond bur at 15,000 rpm under continuous irrigation; (**D**) the two halves obtained after sectioning, showing the exposed pulp cavity from which pulp tissue was retrieved.

Before processing, the tooth (second molar—M2) was photographed in order to document the dental morphology (Figure 1A). Then, the dental element underwent radiographic assessment. A periapical radiograph was acquired to study the root and endodontic morphology to plan the subsequent sectioning and to maximise recovery of pulp tissue from the pulpal compartment. The radiograph was obtained using a Heliodent Plus X-ray unit (Dentsply Sirona, Bensheim, Germany) and a VistaScan size 2 × 3 photostimulable phosphor plate (Dürr Dental, Bietigheim-Bissingen, Germany), with exposure parameters set at 60 kV, 7 mA, and 0.20 s; the plate was subsequently processed and digitised with a VistaScan scanner using VistaScan Ultraview software (version 4.0.13, Dürr Dental, Bietigheim-Bissingen, Germany) (Figure 1B).

Guided by the radiographic findings, the tooth was then sectioned longitudinally along its major axis using a Komet 6911H (104 220) diamond bur (Komet, Lemgo, Germany) at 15,000 rpm under continuous irrigation (Figure 1C).

This procedure allowed the pulp cavity exposure. Pulp tissue was retrieved from both halves of the cavity using sterile excavators (Figure 1D).

#### 2.2.2. DNA Extraction

Genomic DNA was extracted from dental pulp using the QIAamp DNA Mini Kit (Qiagen GmbH, Hilden, Germany), a silica-membrane-based purification method suitable for genomic DNA isolation from tissue specimens. Following the manufacturer’s protocol, pulp tissue was lysed in 200 µL of ATL Buffer supplemented with 20 µL of Proteinase K (20 mg/mL) and incubated at 56 °C for 3 h until complete tissue digestion was achieved. Subsequently, 200 µL of AL Buffer (containing guanidine hydrochloride) was added to the lysate, followed by incubation at 72 °C for 10 min. After addition of 200 µL of absolute ethanol (96–100%) and thorough mixing by vortexing, the entire mixture was loaded onto a QIAamp spin column placed in a 2 mL collection tube and centrifuged at 8000 rpm for 1 min. The membrane was washed sequentially with 500 µL of AW1 buffer (8000 rpm, 1 min) and 500 µL of AW2 buffer (maximum speed, 3 min), with an additional centrifugation at maximum speed for 1 min to ensure complete membrane drying. DNA was eluted by adding 100 µL of AE Buffer to the membrane, incubating at room temperature for 1 min, and centrifuging at 8000 rpm for 1 min.

### 2.3. Method B: Tooth Decalcification Followed by DNA Extraction

#### 2.3.1. Tooth Decontamination and Decalcification

**Figure 2:**
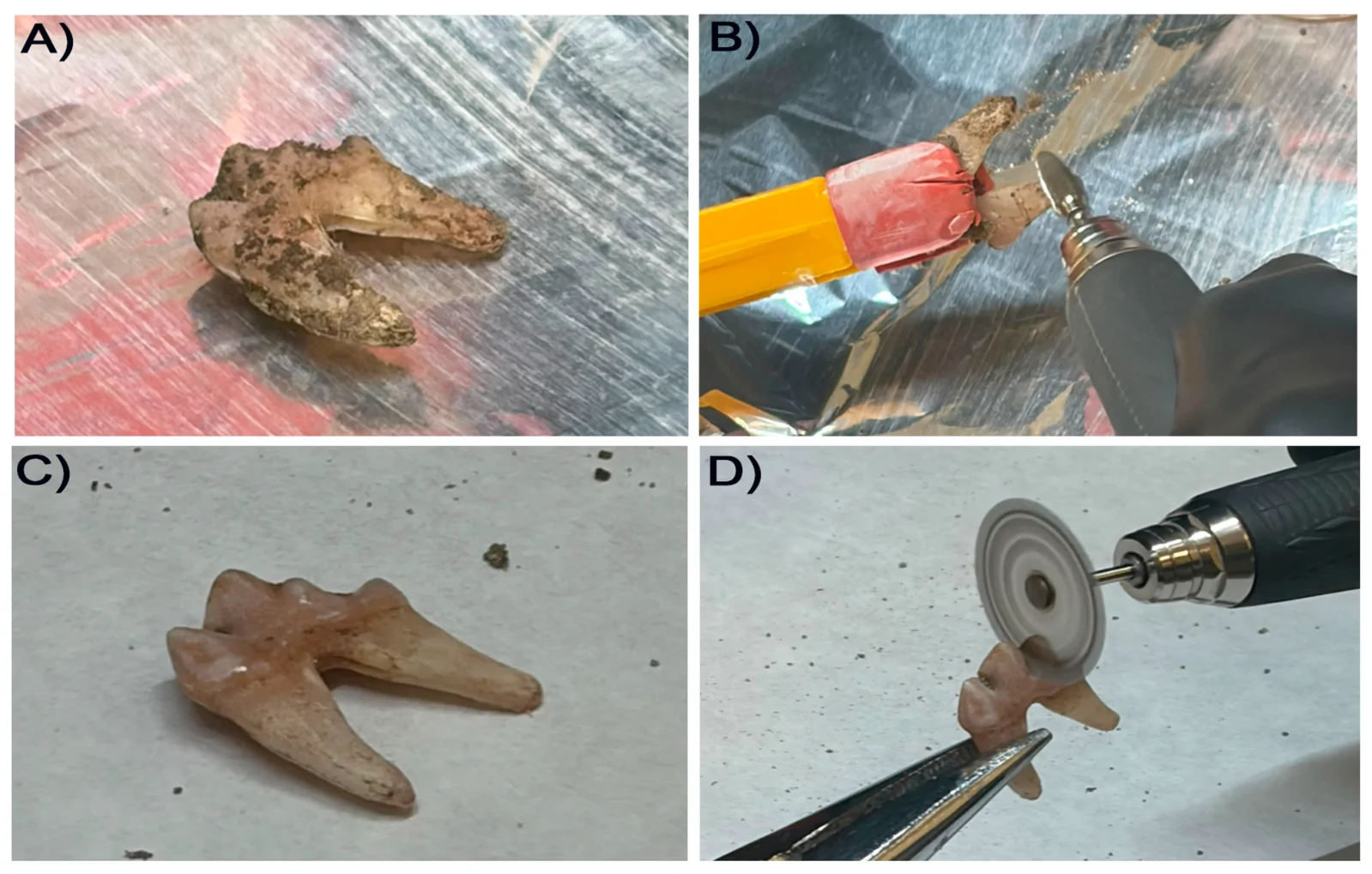
Workflow where the outer surface of the tooth (first molar—M1) (**A**) was cleaned with a rotary dental bur (**B**) to remove surface contaminants (**C**). For decalcification, the tooth was sectioned into fragments using a rotary cutting blade (**D**) as reported in the instructions of the T-Bone Ex kit.

The external surface of the tooth (first molar—M1) was mechanically cleaned using a Dremel rotary dental bur to remove potential surface contaminants (Figure 2A–C). The tooth was subsequently sectioned into 4 fragments of approximately 1 cm × 0.5 cm × 0.5 cm using a rotary cutting blade (Figure 2D).

The commercial T-Bone Ex Kit by DNA Chip Research Inc. (Nakahara-ku, Kawasaki-shi, Kanagawa 211-0004, Japan) [20,21,22] was used for decalcification and lysis according to the manufacturer’s instructions.

Briefly, fragments were immersed in 50 mL of Solution A and incubated at 23 °C for 12 h under continuous agitation, at 450–500 rpm using a thermomixer. After incubation, the temperature was raised to 37 °C, and 1.8 mL of Solution B (pre-warmed to 37 °C for 15 min) was added to Solution A containing the fragments. The mixture was incubated at 37° C for 2 h and shaken at 450–500 rpm with a thermomixer. Following incubation, Solutions A and B were removed. To promote protein digestion, 400 µL of Solution C and 50 µL of Proteinase K were added to a sterile tube containing the decalcified fragments and incubated at 56 °C for 3 h under continuous agitation. After incubation, 500 µL of the digested sample was mixed with 500 µL of TE-saturated phenol and centrifuged at 13,000 rpm for 5 min. The aqueous phase (approximately 400 µL) containing the DNA was recovered and transferred to a sterile microcentrifuge tube (2 mL) for downstream DNA extraction procedures.

#### 2.3.2. DNA Extraction Using EZ2 Connect Instruments

Following the decalcification and pre-lysis steps, DNA extraction was performed using the EZ2 Connect Instrument with Qiagen magnetic bead-based chemistry (Qiagen GmbH, Hilden, Germany), according to the manufacturer’s recommendations. EZ2 Qiagen Kits contain all required reagents and labware for rapid, automated DNA purification using silica-coated magnetic-particle technology, compatible with the new EZ2 Connect instruments. The automated system performs the main steps of DNA purification, including nucleic acid binding, washing, and elution, minimizing manual handling and ensuring standardized processing conditions. Elution was performed using the Qiagen elution buffer supplied with the selected chemistry, and the purified DNA was collected in a final volume of 100 µL and stored at −20 °C until STR analysis.

### 2.4. DNA Extraction of Dam and Offspring

DNA from the dam and offspring was extracted from peripheral blood using the QIAamp Blood Mini Kit (Qiagen GmbH, Hilden, Germany) following the manufacturer’s protocol.

### 2.5. DNA Quantification by Fluorometer

One-microliter aliquots from each sample were tested with the Qubit 4 Fluorometer (Applied Biosystems, Thermo Fisher Scientific, Waltham, MA, USA), using the Qubit™ 1X dsDNA High-Sensitivity (HS) Assay Kit, according to the manufacturer’s protocol.

The fluorescence values automatically give the related DNA concentrations (ng/μL), which are based on the supplied standards used as reference. The assay is selective for double-stranded DNA (dsDNA) and is suitable for quantification of low DNA amounts within the reported detection range of 0.1–120 ng.

### 2.6. PCR Amplification and STR Genotyping

PCR amplification was performed using the Canine Genotypes™ Panel 1.1 Kit (Applied Biosystems, Thermo Fisher Scientific, Waltham, MA, USA), according to the manufacturer’s recommendations. Optimal performance is achieved with 1–2 ng of high-quality genomic DNA in a 20 µL total PCR reaction volume. The kit offers the co-amplification of 18 autosomal microsatellites: AHT121, AHT137, AHTh171, AHTh260, AHTk211, AHTk253, CXX279, FH2054, FH2848, INU055, INU005, INU030, INRA21, REN169O18, REN162C04, REN169D01, REN247M23, REN54P11, and the Amelogenin locus for sex determination.

All samples from the canine family were amplified together with two positive controls: Canine Genotypes Control DNA001, a commercially available genomic DNA at a concentration of 1.0 ng/µL, extracted from an ATCC “MDCK01” canine cell line, and an internal positive control consisting of a canine DNA sample previously genotyped in our laboratory. A negative amplification control containing no DNA was also included in each amplification run.

The same final volume and composition of the PCR reaction mix were used, considering 1 ng of DNA as input, for the controls and Dam and Pup samples.

Since the DNA concentration recovered from the *post-mortem* dental samples was below the optimal range recommended by the manufacturer, the amount of available DNA template was maximized by adjusting only the volume of DNA added to the PCR reaction mix.

For Method A, DNA extract was added at two different volumes (3 µL and 5 µL), corresponding to estimated DNA inputs of approximately 0.3 ng and 0.5 ng in the final PCR reaction volumes (based on a measured DNA concentration of approximately 0.1 ng/µL).

For Method B, 1.7 µL of DNA extract was added to the reaction mix, corresponding to an estimated DNA input of approximately 0.5 ng per reaction (based on a measured DNA concentration of approximately 0.3 ng/µL).

Amplification was performed using a SimpliAmp™ Thermal Cycler (Applied Biosystems, Thermo Fisher Scientific, Waltham, MA, USA), under the cycling conditions recommended for the Canine Genotypes™ assay.

For capillary electrophoresis and fragment analysis, a loading mixture containing 11 µL of Hi-Di™ Formamide and 0.3 µL of GeneScan™ LIZ™ Size Standard per sample was prepared. A total of 2 µL of PCR product was added to the loading mixture. Samples were then denatured and immediately cooled on ice for at least 3 min before being loaded onto the SeqStudio Genetic Analyzer (Applied Biosystems, Thermo Fisher Scientific, Waltham, MA, USA). Fragment analysis and allele calling were performed using GeneMapper^®^ Software v6 (Applied Biosystems, Thermo Fisher Scientific, Waltham, MA, USA). No contamination was detected in the negative control, and the positive controls proved the reaction efficiency and successful conditions for amplifying the known target sequence. To describe the genotype calling, different parameters were evaluated in the analysis of GeneMapper software.

The call rate is a foundational quality control metric that quantifies the success and completeness of automated allele assignment. It reflects the percentage of markers successfully genotyped within a single profile (e.g., a sample call rate of 98% means 49 out of 50 STR loci were successfully resolved).

For each STR locus, the Genotype Quality (GQ) score was calculated as a composite metric (range 0–1) by default threshold settings within the software, integrating marker quality and multiple peak quality values (PQVs) that reflect peak height, shape, binning, and other signal characteristics. Default GQ thresholds were applied: GQ ≥ 0.75 (“Pass”), indicating that the allele assignment was considered robust and reliable; GQ values between 0.25 and 0.75 (“Check”), requiring further evaluation; and GQ < 0.25 (“Low Quality”), requiring manual visual inspection by the operator.

STR loci with a GQ < 0.25 were not automatically excluded. Instead, all STR profiles, including those containing loci below this threshold, were subjected to manual electropherogram inspection by an experienced operator. Final allele assignment was based on the assessment of fluorescence intensity, peak morphology, allele position, and the absence of relevant analytical artefacts.

The overall quality of each sample profile can be summarized by the Composite Genotype Quality (CGQ), a sample-level metric that combines the individual locus-specific GQ values across all typed markers (19 STRs) to provide a global measure of genotype reliability.

The parameters that can drive the reduction in the quality score include the peak height ratio (PHR), the presence of out-of-bin alleles, and altered peak morphology (such as broad peaks).

To evaluate PCR amplification efficiency and signal intensity, the total average peak height considering samples obtained from Method A (higher input) vs. B was calculated by summing the peak heights of all valid alleles across all dye channels, expressed in Relative Fluorescence Units (RFUs), and dividing by the total number of evaluated peaks.

The PHR is a key metric for evaluating intra-locus balance and sample purity. Severe peak imbalance or low PHR values trigger quality flags in GeneMapper.

### 2.7. Mendelian Compatibility and Exclusion Criteria

Mendelian compatibility and parentage assignment were performed by comparison of the 18 autosomal STRs and Amelogenin for sex determination, in accordance with ISAG guidelines for canine parentage testing [23] and given the single-family design of this case report, no population-level statistical analyses were performed.

Each offspring genotype was compared with those of the sire and dam to evaluate Mendelian inheritance consistency. A locus was considered compatible when all alleles observed in the offspring could be unambiguously assigned to either the maternal or paternal genotype. The sire is excluded from parentage when two or more STR incompatibilities are observed between the sire’s profile and the offspring’s obligate paternal allele (OPA) as reported in the ISAG rules [23]. The OPA is defined as the allele in the offspring not attributable to the dam, and it is identified by subtracting maternal contributions from the offspring genotype. When the dam is heterozygous, shared alleles between dam and offspring were assigned to maternal inheritance, while the remaining allele is attributed to paternal origin.

## 3. Results

### 3.1. DNA Concentration from Dental Tissues

DNA extraction produced amplifiable DNA from both dental matrices. Quantification by Qubit showed differences in DNA concentration between the two extraction workflows. Method A yielded a DNA concentration of approximately 0.1 ng/µL, whereas Method B resulted in a concentration of 0.3 ng/µL.

### 3.2. Performance of the Two Methods for Sire STR Genotyping

STR genotyping using the Canine Genotypes™ Panel 1.1 produced 18 autosomal interpretable STR including AHT121, AHT137, AHTh171, AHTh260, AHTk211, AHTk253, CXX279, FH2054, FH2848, INU055, INU005, INU030, INRA21, REN169O18, REN162C04, REN169D01, REN247M23, REN54P11, and Amelogenin for sex determination.

The electropherogram profiles obtained in our analysis show call rates for all genotyped samples, with values of 100% (19/19 loci), except for the sample from Method A, which show a call rate of ~74% (14/19 loci).

The two methods have some operational differences, but taking into account only extracted, PCR amplified and genotyped DNA, Method B revealed a different composite genotype quality (CGQ) than Method A (0.84 vs. 0.33, respectively), indicating a more reliable overall genotypic profile for Method B.

**Figure 3:**
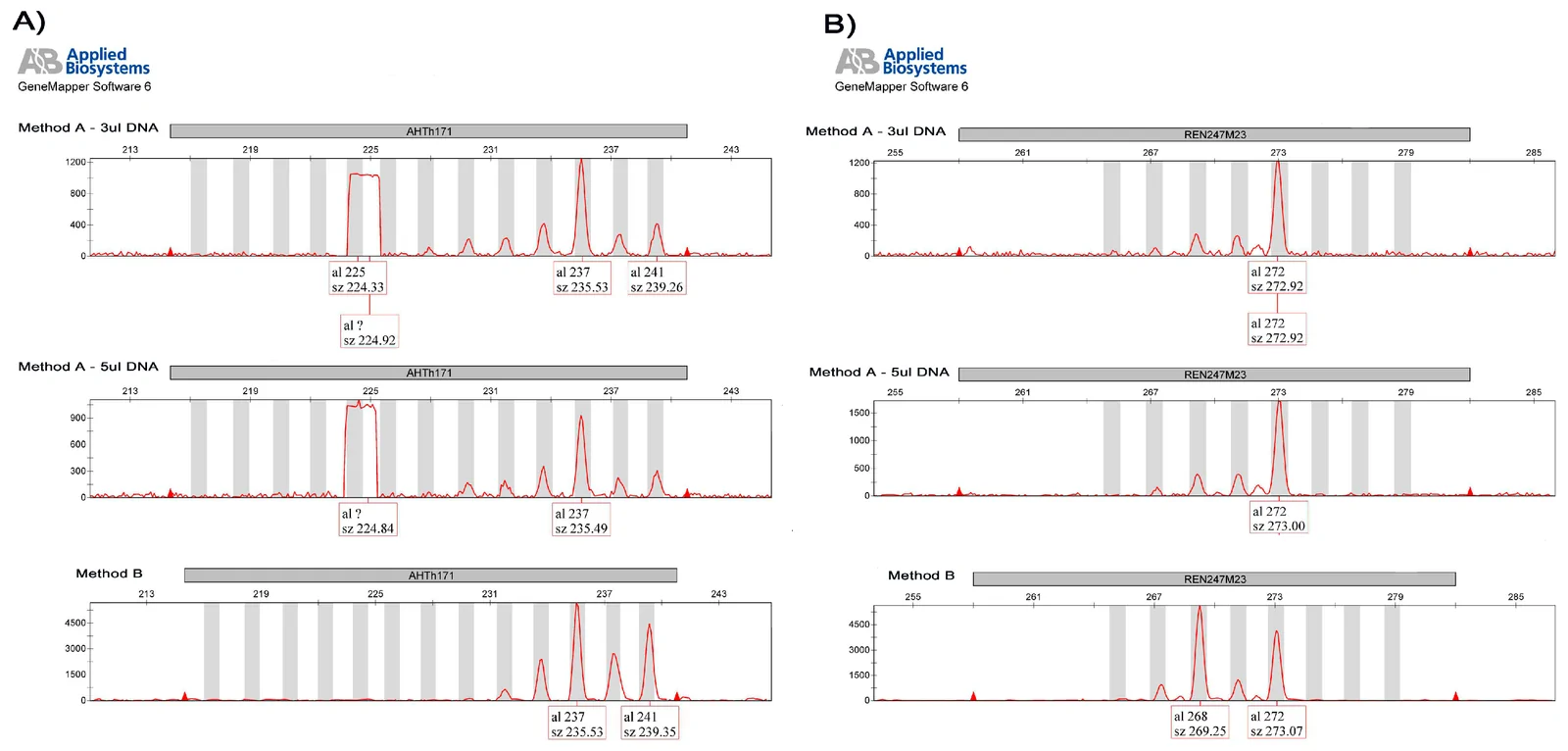
Representative electropherograms illustrating the main issues encountered with the methods. (**A**) At locus AHTh171, Method A showed increased baseline variability, background noise, and artifacts, making profile interpretation more difficult. (**B**) At locus REN247M23, low signal intensity resulted in inconclusive allele calling for some loci, including the one shown.

In fact, in some loci, lower signal intensity was associated with increased baseline variability, and the presence of minor background signals resulted in a difficult interpretation for all alleles required for complete genotyping (Figure 3A).

Several loci exhibited reduced fluorescence intensity and lower peak heights than those observed with Method B (Supplementary Table S1). The mean peak height was 654.88 RFUs for Method A and 5050.08 RFUs for Method B. In Method B, peak morphology was more defined, with sharper allelic peaks and reduced background noise throughout the electropherograms.

The baseline remained stable across the size range analyzed and non-specific signals were reduced, with genotype quality achieved via GeneMapper^®^ Software v6 (Applied Biosystems, Thermo Fisher Scientific, Waltham, MA, USA) exceeding the predetermined threshold or sufficient to allow, where necessary, a manual analysis for the interpretation of the allele in the analysis.

The peak height ratio that represents the relationship between the two alleles at the heterozygous locus shows a consistent value for all the loci in Method B and passes the quality control cutoff of the software.

Allele calls were inconclusive for 5 microsatellite markers in input samples obtained by Method A. A compromised amplification directly associated with a partial genotype interpretation was observed in microsatellites: AHTh171, AHTk211, INU005, REN169D01 and REN247M23 (Figure 3B). These dinucleotide repeats are located on chromosomes 6, 26, 33, 14 and 15, respectively. They exhibit size ranges of 212–239 bp, 79–101 bp, 102–136 bp, 199–221 bp, and 258–282 bp, and are labelled with distinct fluorochromes (red, blue, black, and green) (Table 1).

### 3.3. Mendelian Compatibility and Exclusion Criteria

Since the analysis involved a single-family case, the interpretation was based on Mendelian compatibility and ISAG guidelines for canine parentage assignment rather than population-based statistical parameters.

Method B was selected for parentage assignment because it generated a complete and interpretable STR profile suitable for allele comparison. The STR profile of the sire obtained using Method B was compared with the maternal and offspring profiles to evaluate Mendelian compatibility (Table 1). All 18 autosomal STR loci analysed showed Mendelian compatibility between the tested sire, the dam, and the three offspring. No incompatibilities or exclusion events were identified across the marker panel (Table 1). For all informative STRs (AHT121, AHTh171, AHTk211, AHTk253, FH2054, INU055, INU005, INU030, INRA21, REN169D01 and REN247M23), the obligate paternal alleles (OPAs) could be consistently inferred after subtraction of the maternal contribution, and all reduced paternal alleles were compatible with the tested sire profile. Therefore, based on the available informative STR data, the tested male was not excluded as the biological sire of the three offspring, and the genetic profiles were consistent with the alleged parentage relationship.

## 4. Discussion

Canine forensic DNA analysis has emerged as a valuable tool in different contexts of application, but the field continues to face significant challenges, including methodological issues, with limited standardization of technical aspects and validation of protocols, and persistent gaps in quality control and supporting resources [24,25].

The present study addresses a realistic forensic veterinary scenario in which the only available biological material for the genetic identification of a deceased registered dog was *post-mortem* dental tissue, as reported for the Tudor dog recovered from the Mary Rose wreck and in the ancient remains [13,26,27].

The quality, consistency, and recovery of DNA at the extraction stage play a critical role in determining the reliability of results, especially in genotyping applications [28].

Within this case, two DNA extraction workflows were applied to different dental matrices to assess their suitability for recovering DNA of sufficient quality for STR-based parentage testing and to provide practical methodological information that may assist similar forensic investigations [29].

Despite several workflows recommending combining spectrophotometry with fluorometry when enough material is available, to obtain both purity indices and dsDNA concentration, forensic samples are frequently characterized by low template DNA and possible degradation, so we chose fluorometric quantification with the Qubit dsDNA HS assay rather than relying on UV spectrophotometry [30]. The literature shows that fluorometric methods are more sensitive and more specific for dsDNA, while spectrophotometric measurements become less accurate at low concentrations, not providing complete information on DNA purity or integrity [31,32].

As described above, two different methods were employed in this case report. The two approaches differ in several aspects, primarily because they are based on different starting matrices (dental pulp vs. tooth fragments), require different processing times (with Method B requiring considerably longer laboratory processing times), and involve different chemical such as phenol.

Overall, both methods allowed DNA extraction and subsequent PCR amplification, resulting in the generation of STR profiles, but the quality and completeness of the profiles obtained differed between the two approaches.

A detailed analysis of the results obtained with the two methods reveals that the higher CGQ observed for Method B suggests that this approach produced more robust and consistent genotype calls across the analysed STR loci. In contrast, the lower CGQ associated with Method A may reflect reduced signal quality and/or a greater number of loci that require manual review or yield low-quality genotype calls. However, because CGQ is a composite sample-level metric, locus-specific GQ values and electropherograms should be examined to identify the markers directly and consider the difference in the profiles (Supplementary Table S1).

The call rate for Method A is under 75%, resulting in 5 inconclusive loci out of 19 tested and these pose challenges for the automatic annotation by the analysis software and for the accurate interpretation by the operators, especially for AHTh171, INU005, REN169D01 and REN247M23; instead, the inconclusive call on the AHTk211 marker is related to the high background noise present. These target regions generate different size ranges and are labelled with different fluorescent dyes (Supplementary Table S1).

The electropherograms produced by Method A showed a total lower fluorescence intensity, with more than 7 times less average peak height. As reported in the representative electropherograms in Figure 3, the lack of automatic annotation for these loci are related to a signal that was less clearly defined. Increased baseline irregularities and the undesired occurrence of non-specific amplification products complicated data interpretation, with also high differences in fluorescence intensity of heterozygous peaks.

Taken together, these findings suggest that Method A yielded substantially weaker signal intensities across multiple loci, which may indicate less efficient amplification/detection compared with Method B. This difference has important implications not only on the reliability of downstream fragment analysis but also for genotype misclassification. In particular, the analysis highlights that, when using Method A, careful evaluation of peak morphology, including height, profile, and relative intensity, is required to reliably distinguish true alleles from artefactual signals or non-specific amplification products, which may otherwise compromise genotype interpretation. In this case, the results obtained with Method B facilitated the correct identification of the true alleles, preventing inconclusive or partial final results.

This observation suggests that the technical limitation likely resides in the quantity and/or quality of the input DNA.

We hypothesize that the lower signal quality observed in the electropherograms obtained from Method A may be associated with the use of dental pulp as starting material, which may contain a limited amount of recoverable nucleated cellular material and may be more susceptible to *post-mortem* degradation. It should be acknowledged that the two workflows described were applied to different dental matrices: Method A was performed exclusively on dental pulp, whereas Method B was applied to decalcified tooth fragments containing dentin, cementum, and potentially residual pulp tissue. Therefore, the observed differences in STR-based genotyping performance cannot be attributed exclusively to the extraction procedures, as they may also reflect intrinsic differences in the biological substrates analysed, as suggested.

However, because this study involved a single deceased animal and two extraction workflows applied to different dental substrates, the observed differences in electropherogram quality cannot be attributed exclusively to the extraction workflow.

We must also underscore the intrinsic limitations of using predominantly dinucleotide STR markers such as Canine Genotypes™ Panel 1.1. In particular, dinucleotide STRs are known to generate higher stutter artefacts than markers with longer repeat motifs (tetranucleotide and pentanucleotide), which can complicate allele calling, reduce automation robustness, and make profile interpretation more challenging, especially in low-template or mixed samples [33,34].

Another limitation of the present case report is the lack of statistical evaluation with a larger sample size. Since the analysis involved a single-family case, the interpretation was based on Mendelian compatibility and ISAG guidelines for canine parentage assignment rather than population-based statistical parameters.

Furthermore, it is important to note that, although all appropriate laboratory procedures were followed to minimize the risk of contamination during the DNA extraction process, the absence of negative control during the extraction phase may represent a limitation of the study. This limitation is particularly relevant in a forensic context, where the low quantity of DNA and the potential for contamination require stringent contamination-control measures.

Taking these considerations into account, we tested that both approaches allowed the recovery of informative STR profiles suitable for parentage assignment using the widely validated Canine Genotypes™ Panel 1.1. The findings reported in this case highlight the importance of considering the type, composition, and preservation status of available dental tissues when planning DNA recovery strategies in *post-mortem* canine samples. This case report provides practical methodological information that may assist forensic investigations in which dental remains represent the only available biological material for genetic identification.

## 5. Conclusions

The present case report describes STR genotyping from DNA samples successfully recovered from *post-mortem* canine dental tissues. The application of two DNA extraction workflows using different dental matrices provided practical technical information in a realistic forensic veterinary scenario where teeth represented the only available biological material. Although both approaches generated usable genetic profiles, the decalcification-based workflow (Method B) produced electropherograms characterized by higher signal intensity, reduced background noise, and improved allele resolution, leading to conclusive genotyping.

Therefore, based on the available STR data, the tested male was not excluded as the biological sire of the three offspring, and the genetic profiles were consistent with the alleged parentage relationship. Overall, this case highlights the forensic value of *post-mortem* canine teeth as a source of DNA for genetic identification and parentage testing, while providing practical methodological insights that may assist similar forensic investigations in which dental tissue is the only biological matrix available.

## Figures and Tables

**Table 1:** The STR profiles reporting the paternal genotype from DNA obtained using Method B (decalcification followed by DNA extraction), the maternal genotype and the three offspring. The parentage assessment in the offspring is compared with the father’s STR profile.

Locus	Size Range *^,1^	Motif	Dye Color ^2^	Sire *	Dam *	Pup 1 *	Pup 2 *	Pup 3 *	OPA **
AHT121	68–118	di	Red	100/106	96/106	96/100	106	100/106	Yes
AHT137	126–156	di	Green	147	147/149	147/149	147/149	147/149	No
AHTh171	212–239	di	Red	237/241	235/241	237/241	241	241	Yes
AHTh260	230–254	di	Green	244/246	240/246	246	240/246	240/246	No
AHTk211	79–101	di	Blue	91/95	95	91/95	95	95	Yes
AHTk253	277–297	di	Green	284/292	288/292	292	284/292	292	Yes
CXX279	109–133	di	Blue	116	116	116	116	116	No
FH2054	135–179	tetra	Red	176	172	172/176	172/176	172/176	Yes
FH2848	222–244	di	Black	232/240	232/240	232/240	240	232	No
INU055	190–216	di	Blue	210/212	210	210/212	210/212	210	Yes
INU005	102–136	di	Black	124/128	124	124/128	124/128	124/128	Yes
INU030	139–157	di	Black	144/152	144	144	144/152	144	Yes
INRA21	87–111	di	Green	97/101	97/103	97/103	97/101	97	Yes
REN169O18	150–170	di	Blue	168	164/168	164/168	168	168	No
REN162C04	192–212	di	Red	202	202	202	202	202	No
REN169D01	199–221	di	Green	202/210	202/218	202/218	202/210	202/218	Yes
REN247M23	258–282	di	Red	268/272	272/280	268/272	268/272	272/280	Yes
REN54P11	222–244	di	Blue	222/232	222/232	232	222	222	No
Amelogenin	174–218	-	Black	XY	XX	XY	XX	XY	-

* 18 autosomal STRs are reported in base pairs (bps), Amelogenin marker is reported as X/Y. ** Obligate Paternal Allele. ^1^ Size ranges are based on information provided by ISAG and data generated by Applied Biosystems, Thermo Fisher Scientific, Waltham, MA, USA. The data represents a large selection of dog breeds. However, some breeds may have alleles outside the ranges provided. ^2^ Dye colors are listed as they appear following electrophoresis with Filter Set G5 (Applied Biosystems, Thermo Fisher Scientific, Waltham, MA, USA).

## Data Availability

The original contributions presented in this study are included in the article/Supplementary Material. Further inquiries can be directed to the corresponding authors.

## Supplementary Materials

The following supporting information can be downloaded at https://www.mdpi.com/article/10.3390/vetsci13090948/s1, Table S1. STR profiles automatically assigned by the software Gene Mapper.xlsx.
